# Gout and Risk of Myocardial Infarction: A Systematic Review and Meta-Analysis of Cohort Studies

**DOI:** 10.1371/journal.pone.0134088

**Published:** 2015-07-31

**Authors:** Shuang-Chun Liu, Lei Xia, Jin Zhang, Xue-Hong Lu, Da-Kang Hu, Hai-Tao Zhang, Hai-Jun Li

**Affiliations:** 1 Department of Clinical Laboratory, Taizhou Municipal Hospital, Taizhou, Zhejiang, China; 2 Department of Urinary Surgery, Affiliated Renji Hospital, Medical School of Shanghai Jiao Tong University, Shanghai, China; 3 Department of Pediatrics, Taizhou Municipal Hospital, Taizhou, Zhejiang, China; 4 Department of Urinary Surgery, Taizhou Municipal Hospital,Taizhou, Zhejiang, China; 5 Department of Neurology, Taizhou Municipal Hospital, Taizhou, Zhejiang, China; University of Bologna, ITALY

## Abstract

**Introduction:**

A high incidence of myocardial infarction among patients with gout has been suggested by several observational studies. We performed a meta-analysis to evaluate the association between gout and the risk of myocardial infarction.

**Materials and Methods:**

The PubMed and Embase databases were searched from inception to October 2014 for cohort studies that evaluating the association between gout and the risk of myocardial infarction. Summary estimates were derived using a random-effects model and reported as relative risks (RRs) with 95% confidence intervals (CIs).

**Results:**

Five studies involving 8,656,413 participants with a total of 1000 MI events were included. Overall, gout was associated with an increased risk of myocardial infarction (RR 1.45; 95% CI, 1.19–1.75; *p*<0.001), and the association referred to non-fatal myocardial infarction (RR 1.29; 95% CI, 1.19–1.39; *p* <0.001) but not fatal myocardial infarction (RR 1.11; 95% CI, 0.96–1.28; *p* = 0.174). The increased risk was observed in both women (RR 1.62; 95% CI, 1.18–2.21; *p* = 0.003) and men (RR 1.45; 95% CI, 1.21–1.74; *p* <0.001). Stratified analysis revealed a gradual increase in myocardial infarction risk with a younger age of gout onset (age 20–44 years old (RR 2.82; 95% CI, 1.38–5.79; *p* = 0.05); 45–69 years old (RR 1.85; 95% CI, 1.22–2.82; *p* = 0.04); ≥70 years old (RR 1.52; 95% CI, 1.22–1.88; *p* <0.001)).

**Conclusion:**

This meta-analysis suggests that patients with gout have an increased risk of myocardial infarction.

## Introduction

According to World Health Organization estimates, ischemic heart disease is the primary cause of death worldwide, accounting for 7.4 million fatalities [1,2]. Myocardial infarction (MI), which accounts for the majority of ischemic heart disease cases and affects approximately 1 US citizen per 44 seconds, is associated with undesirably high rates of morbidity and mortality; approximately 15% of MI cases are fatal [3,4]. Therefore, an exploration of MI risk factors for primary prevention should be a major public health priority.

Gout is the most prevalent form of inflammatory arthritis in developed countries, the prevalence of gout in clinical practices in the UK and Germany is 1.4%, and this disease is estimated to affect 0.9% and 3.9% adults in France and the USA respectively [5–7]. Epidemiologic studies have indicated that the incidence and prevalence of gout are increasing worldwide and that more people are at an increased risk of developing gout [8–10]. In gout patients, the inflammatory response associated with gout plays a key role in the initiation and progression of atherosclerosis, and promotion of a pro-thrombotic environment that leads to acute coronary events such as angina or MI [11,12]. To test this important hypothesis, a number of observational studies during the past decade have addressed the association between gout and the risk of MI [13–19]. However, no systematic reviews or data pooling for a definite conclusion have been attempted; we therefore performed a meta-analysis of cohort studies to evaluate the risk of MI among patients with gout versus those without gout.

## Materials and Methods

### Literature search

We adopted the Meta-analysis Of Observational Studies in Epidemiology (MOOSE) checklist when reporting this review [20]. We systematically searched the PubMed and Embase databases (from inception to October 2014) for studies investigating the association between gout and myocardial infarction. No language restrictions were imposed. Search terms included ‘gout’ and ‘myocardial infarction’. Furthermore, the reference lists of identified manuscripts were hand-searched and scrutinized to identify other relevant publications.

### Selection criteria

We applied the following inclusion criteria when selecting eligible studies: (i) cohort study; (ii) examination of the association between gout and the risk of MI; (iii) reported the effect size data such as odds ratios (ORs), hazard ratios (HRs) or relative risks (RRs) with 95% confidence intervals (CIs) or provided data allowing the calculation of these values.

### Data extraction

The following information were extracted from each included study: first author, publication year, country, population characteristics, cohort size, number of gout cases, number of MI events, follow-up duration, ascertainment of gout, assessment of MI, and adjusted covariates. Supplementary files were also examined for data abstraction. When necessary, we contacted the authors of included articles for additional data. Extracted data were entered into a standardized Excel (Microsoft Corporation, Redmond, WA, USA) file. Data extraction was performed independently by two authors (SCL and LX). Any disagreement was resolved by discussion and consensus.

### Assessment of study quality

The Newcastle–Ottawa Scale (NOS) was used to assess the quality of each included study [21]. The quality of cohort studies was evaluated according to the following three major components: selection of the study group (0–4 points); quality of the confounding adjustment (0–2 points); and assessment of cohort outcomes (0–3 points). A higher NOS score indicates better methodological quality, as well as a lower risk of bias.

### Statistical analysis

We used RR as the common measure of an association between gout and the risk of MI. We conducted additional analyses to evaluate the risks of fatal MI and non-fatal MI, as well as the risks in both women and men. Furthermore, we performed stratified analyses to evaluate differences in risk between age groups (20–44 years versus 45–69 years versus ≥70 years). Heterogeneity was assessed using the Cochran Q statistic (*p* <0.1) and measured with the *I* ^*2*^ statistic, which was used to estimate the percentage of effect size variability that is attributable to heterogeneity across studies [22–24]. *I²* values of 25%, 50%, and 75% were used as cut-off points for low, moderate, and significant degrees of heterogeneity respectively. A random-effects model was used regardless of heterogeneity. A *p* value <0.05 was considered statistically significant, unless where otherwise specified. We used STATA, version 12.0 (Stata Corporation, College Station, TX, USA) to perform the statistical analysis.

## Results

### Description of the studies

**Fig 1:**
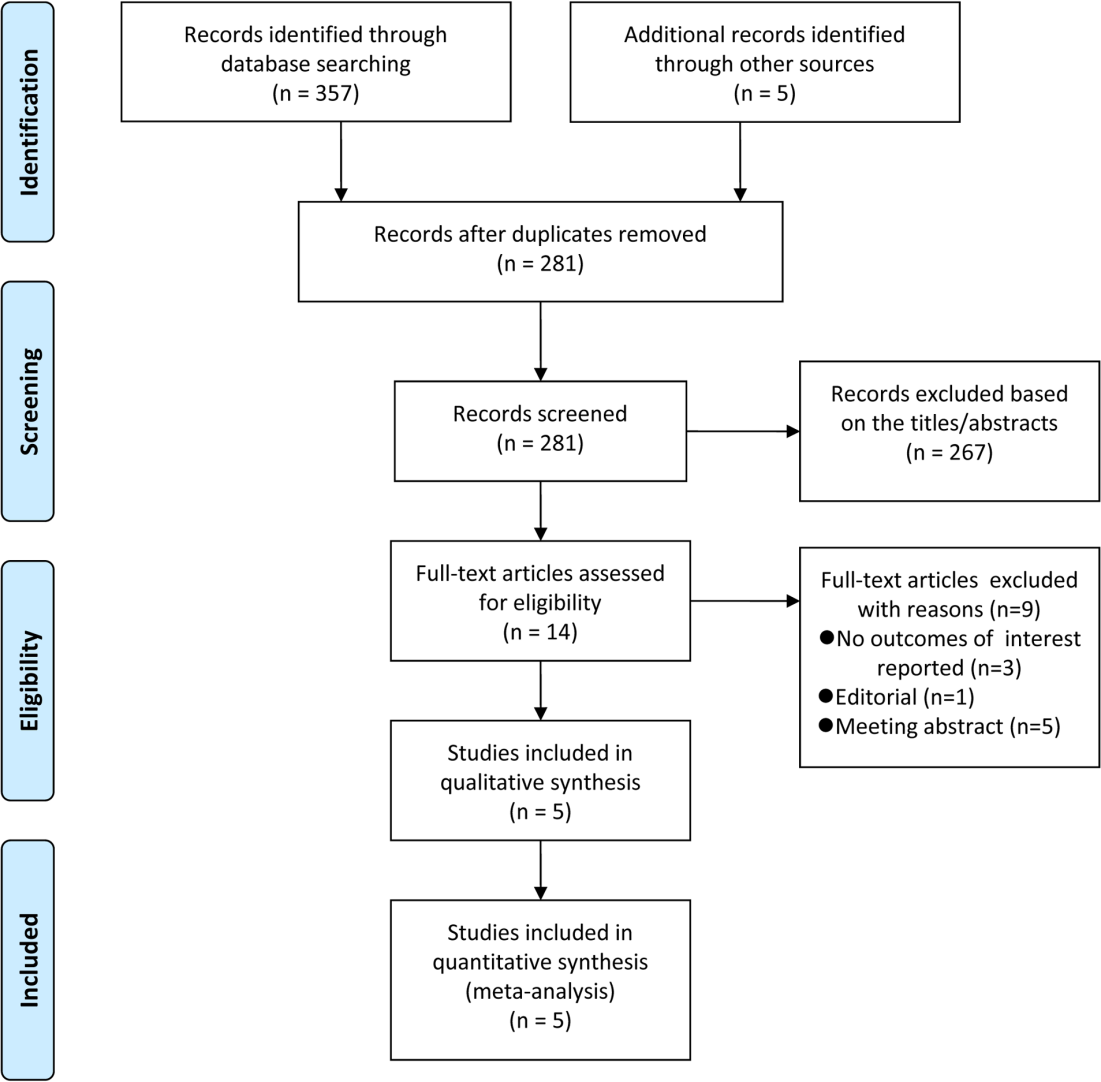
Flow diagram for selection of articles.

A detailed flowchart of the search and selection results is shown in Fig 1. The initial search identified a total of 357 potentially relevant records through electronic searching, and five additional records identified through other sources. Eighty-one records were excluded as duplicates, 267 were identified as irrelevant after reading the titles and abstracts, and the remaining 14 full-text articles were eligible for further assessment. After the inclusion criteria were applied, five studies with a total of 8,656,413 participants were included in the meta-analysis [13–17].

Table 1 shows the major characteristics of the five eligible cohort studies (additional details have been provided in S1 Table.), which were published between 2006 and 2013. Two studies were conducted in Canada, and one study each was conducted in the USA, UK, and China. The numbers of participants in each study ranged from 1,175 to 7,357,019 and the mean follow-ups ranged from 3.8 to 12 years. Study-specific quality assessments scores for the five articles are summarized in S2 Table. The quality scores ranged from 7 to 9 with an average score of 8, indicating that all the cohort studies are of high quality.

**Table 1:** Main characteristics of cohort studies included in the meta-analysis. MI: myocardial infarction, AMI: acute myocardial infarction, PC: prospective cohort, RC: retrospective cohort, NA: not available, CABG: coronary artery bypass graft, ECG: electrocardiogram.

Author/year	Category	Area	Age (mean), years	Man, %	Quality	Study size	Gout	MI	Follow up, years	Study design	Definition of gout	Assessment of MI
Krishnan/2006 [13]	NA	USA	Gout 47 Not gout 46	100	9	12,866	1123	1108	6.5	PC	Self-report+documentation	Physician evaluations, hospital records, EKGs, and CABG surgery.
Choi/2007 [14]	Confirmed	Canada	Gout 59 Not gout 54	100	8	1175	23	NA	12	PC	American College of Rheumatology survey criteria	Questionnaires and medical record
Choi/2007 [14]	Self-reported	Canada	Gout 59 Not gout 54	100	7	1175	53	NA	12	PC	Self-reported	Questionnaires and medical record
DeVera/2010 [15]	Men	Canada	Gout 73.9 Not gout 73.3	100	8	34512	5752	2272	7	RC	ICD-9	ICD-9
De Vera/2010 [15]	Women	Canada	Gout 75.0 Not gout 75.0	0	8	23340	3890	996	7	RC	ICD-9	ICD-9
Kuo/2013 [16]	Men	China	NA	100	8	360432	18674	2636	8	RC	ICD-9	ICD-9
Kuo/2013 [16]	Women	China	NA	0	8	344071	7882	1082	8	RC	ICD-9	ICD-9
Seminog/2013 [17]	England	UK	NA	74	7	7357019	202033	10995	3.8	RC	ICD-7,8,9,10	ICD-10
Seminog/2013 [17]	ORLS	UK	NA	73	8	521823	3174	89	5.7	RC	ICD-7,8,9,10	ICD-10

### Meta-analysis

#### Gout and risk of MI

**Fig 2:**
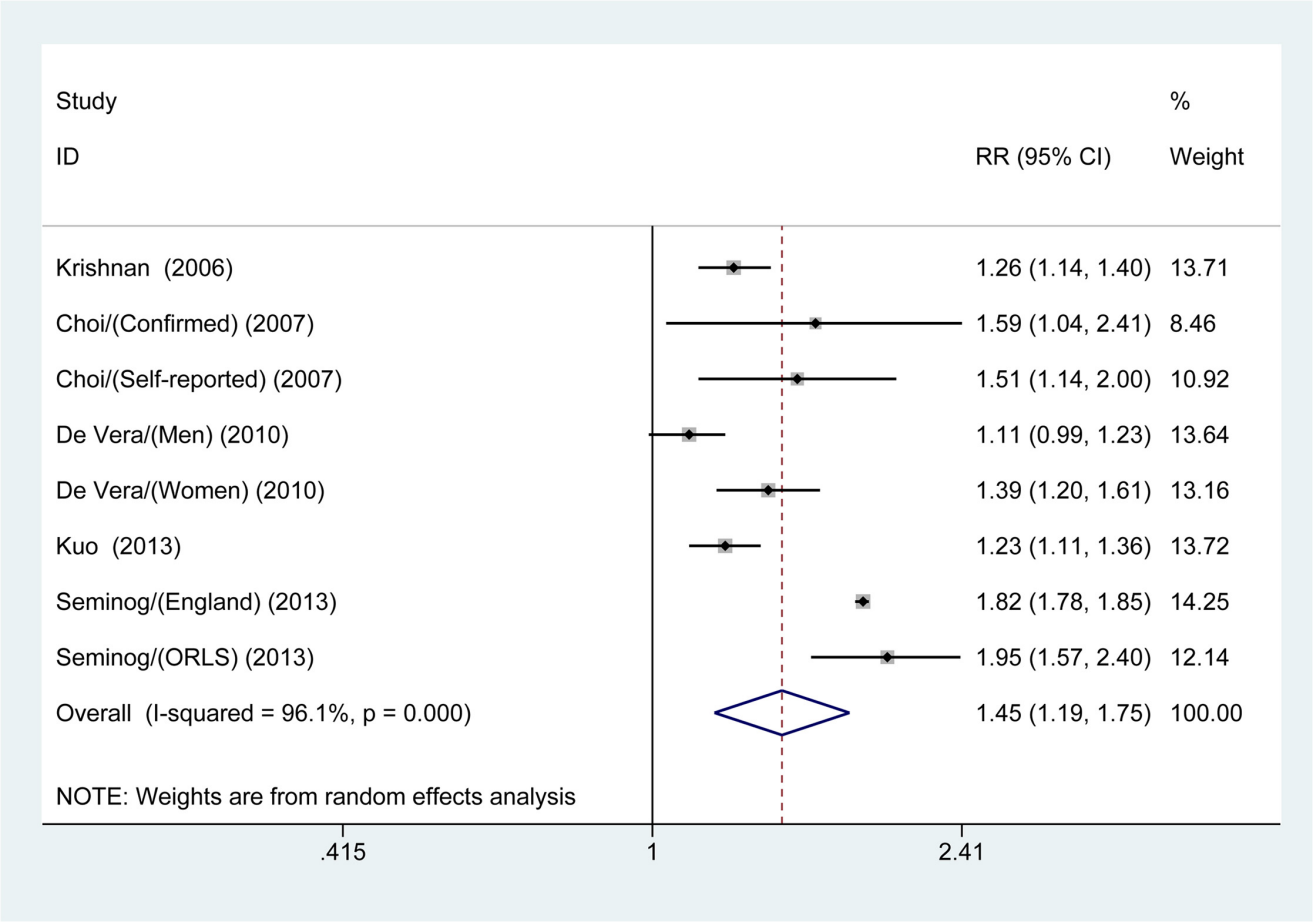
Forest plot showing the risk of myocardial infarction in patients with gout.

Fig 2 shows the pooled results from the random-effects model that combined the multivariable-adjusted RRs for MI. A total of 8,656,413 participants were included in the analysis (242,604 cases with gout versus 8,413,809 cases without gout). Patients with gout had an increased risk of MI (RR 1.45; 95% CI, 1.19–1.75; *p* <0.001), and significant heterogeneity was observed (*I* ^*2*^ = 96.1%; *p* <0.001).

Because of variations between studies with respect to use of the categories of MI, age and sex used in risk reporting, it was impossible to combine all five included studies and calculate a summary RR for each category.

#### Stratified analyses

**Fig 3:**
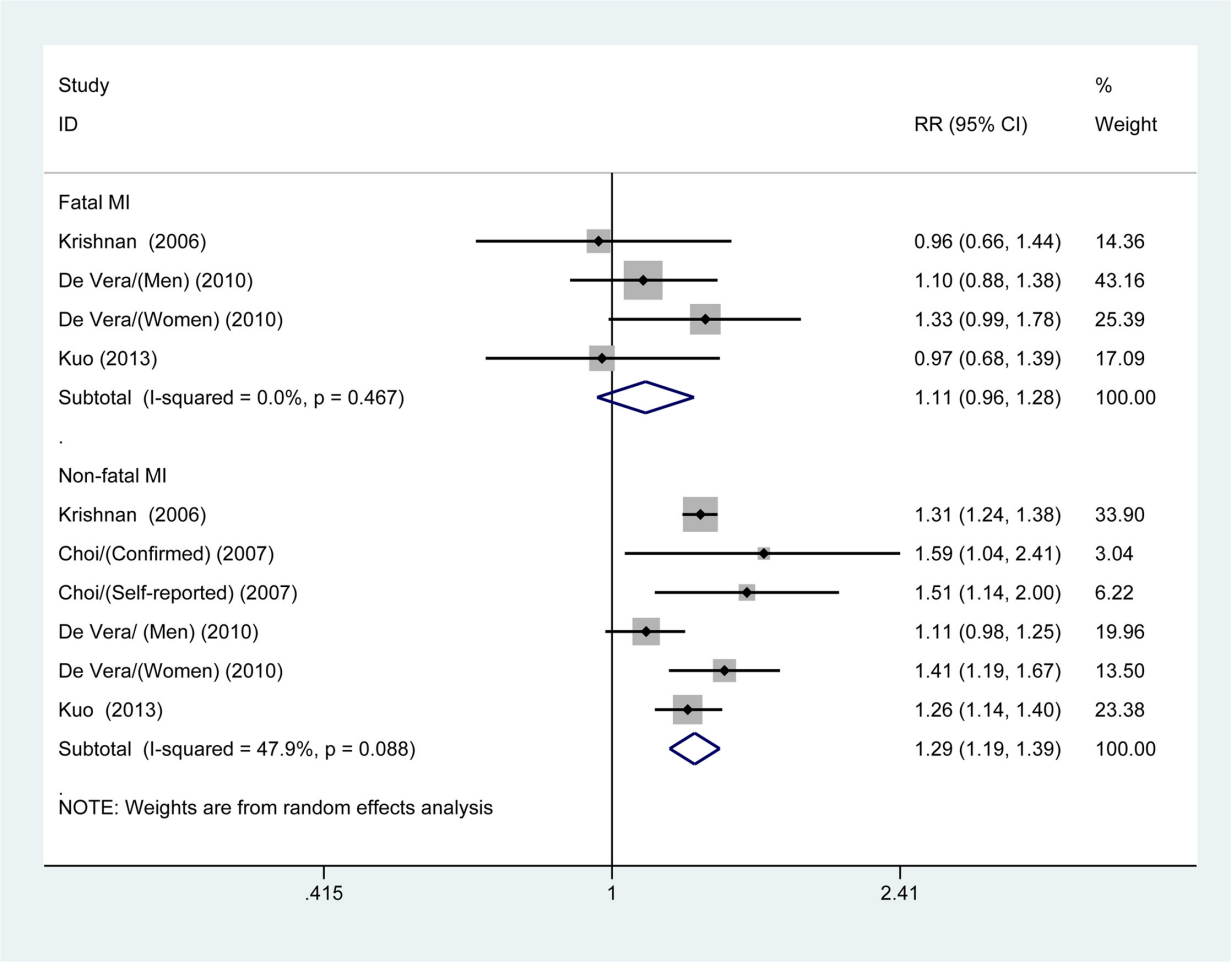
Meta-analysis of the association between gout and different kinds of myocardial infarction (MI; fatal MI and non-fatal MI).

**Fig 4:**
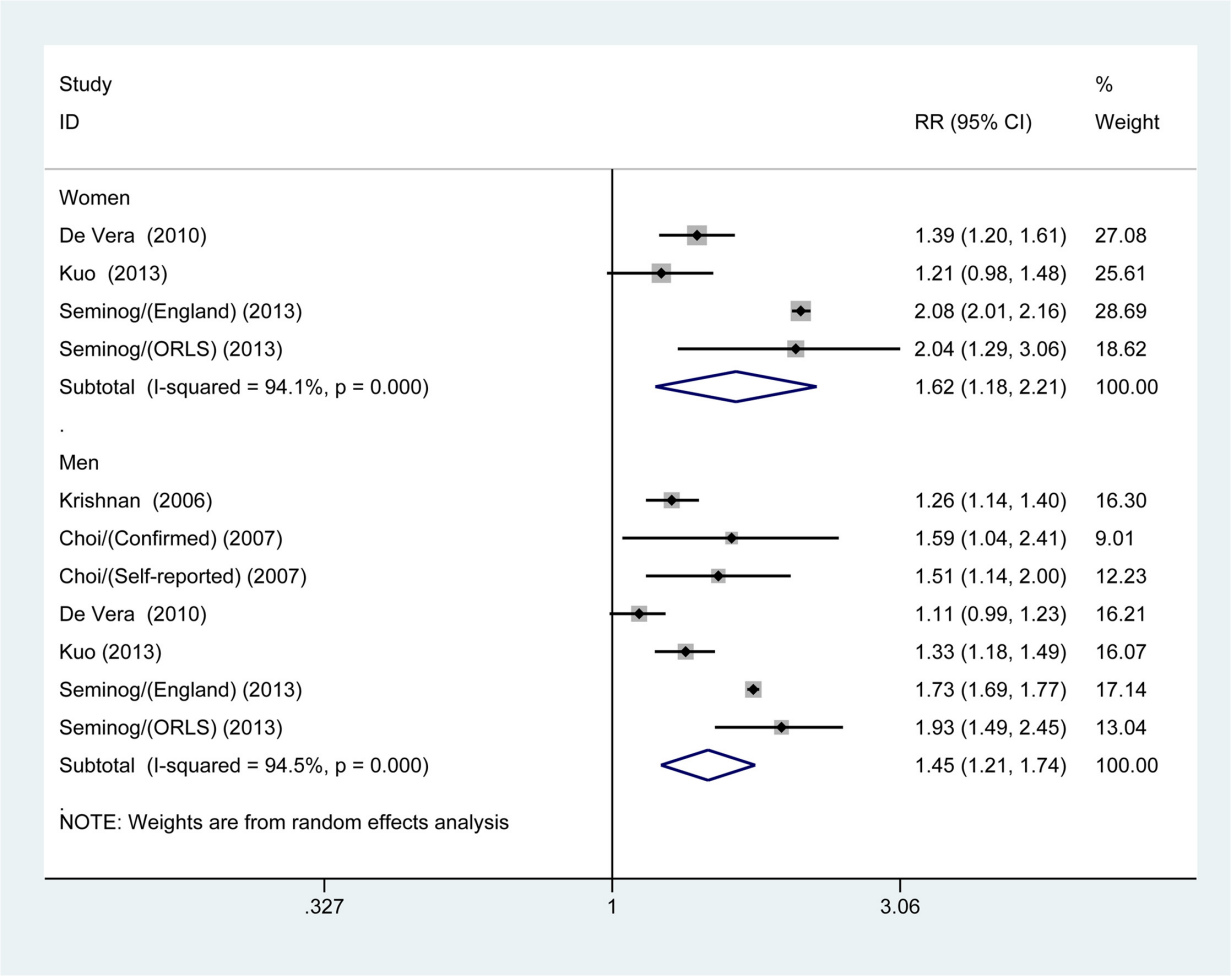
Meta-analysis of the association between gout and risk of myocardial infarction in women and men.

The multivariable-adjusted RRs of combined appropriate studies for both fatal MI and non-fatal MI are shown in Fig 3. The pooled estimates of multivariable-adjusted RRs identified an apparent increased risk of non-fatal MI in gout patients (RR 1.29; 95% CI, 1.19–1.39; *p* <0.001); however, we found no significant association between gout and risk of fatal MI (RR 1.11; 95% CI, 0.96–1.28; *p* = 0.174). We additionally performed a stratified analysis according to gender among patients with gout; the pooled estimated of multivariable-adjusted RRs for the risk of MI were (RR 1.62; 95% CI, 1.18–2.21; *p* = 0.003) for female patients with gout and (RR 1.45; 95% CI, 1.21–1.74; *p* <0.001) for male patients, respectively (Fig 4).

**Fig 5:**
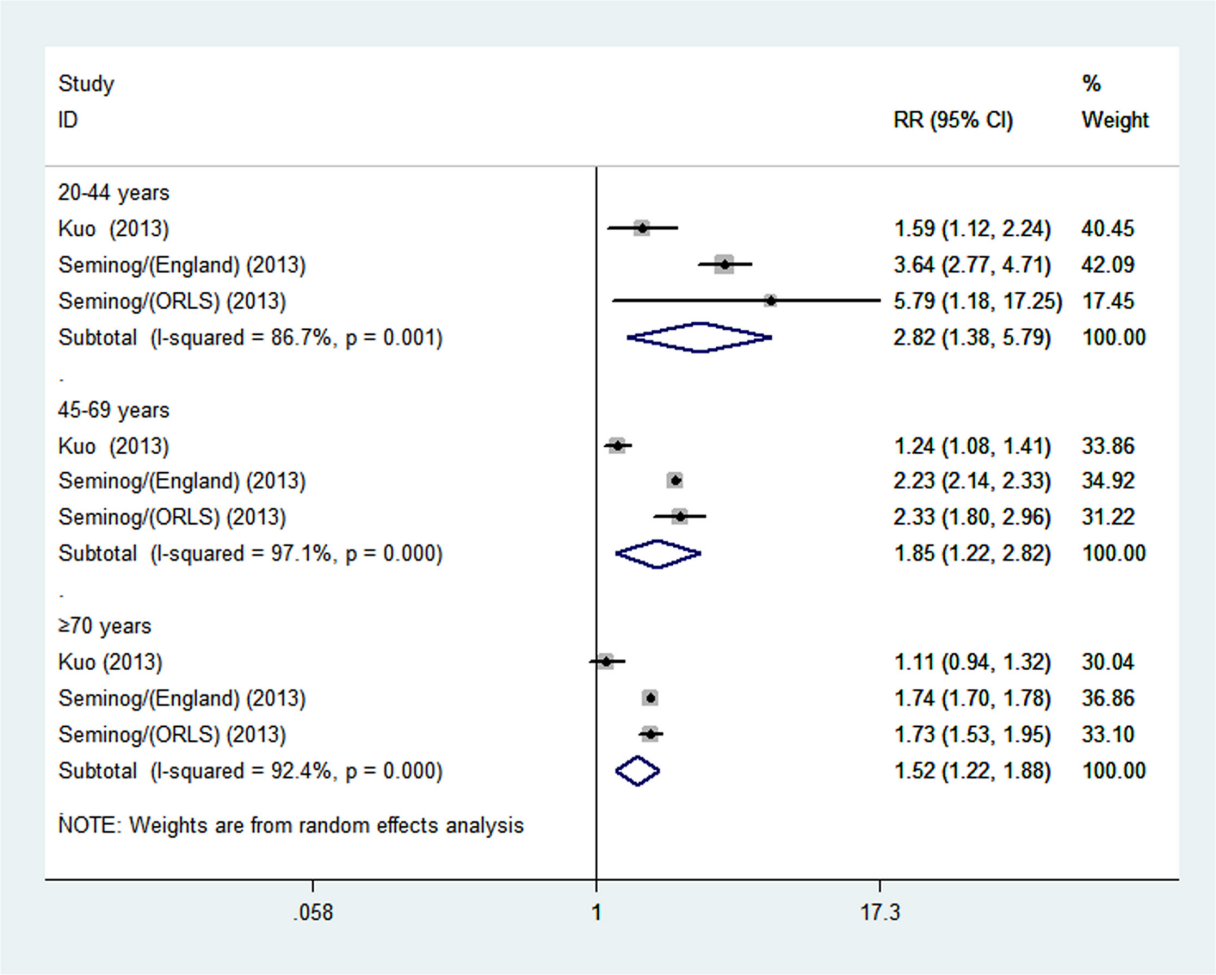
Meta-analysis of the association between gout and risk of myocardial infarction in age groups.

Through a comparison of individuals with and without gout in each age group, we observed a gradual increase in MI risk with a younger age at gout onset. As shown in Fig 5, the pooled results demonstrated this graduated relationship: in the 20–44 years age group (RR 2.82; 95% CI, 1.38–5.79; *p* = 0.05); in the 45–69 years age group (RR 1.85; 95% CI, 1.22–2.82; *p* = 0.04); and in the ≥70 years age group (RR 1.52; 95% CI, 1.22–1.88; *p* <0.001).

## Discussion

### Main findings

To the best of our knowledge, this is the first systematic review and meta-analysis to investigate the association between gout and the risk of MI. The pooled results of five cohort studies suggest that both male and female patients with gout have an increased risk of non-fatal MI, but not of fatal MI. Furthermore, stratified analyses revealed a gradient of increased MI risk with a younger age at gout onset.

### Possible mechanisms

Although our meta-analysis indicated that patients with gout have an increased risk of MI, the underlying mechanism remains unclear and there are several possible mechanisms might explain the observed association. Hyperuricemia, which causes gout pathogenesis, and uric acid, which perpetrates hyperuricemia, have been found to associate with cardiovascular disease (CVD).

The inflammatory response associated with gout is characterized simultaneously by the initiation of an acute attack and other typical complex interactions between various cell types [25]. Inflammation associated with gout may play an important role in the initiation and progression of atherosclerosis, as well as in plaque disruption and thrombotic complications, and the various triggers, amplifiers and innate and adaptive immune responses in the cascade of inflammatory events that promotes atherogenesis and thrombogenesis in correlation with CVD. And inflammation also has a long-term prognostic value because inflammation after CVD is associated with an increased risk of recurrent coronary events [26–31]. Therefore, the complex mechanisms of inflammation in patients with gout may lead to an increased risk of CVD.

Hyperuricemia is known to associate with various risk factors of cardiac disease, such as hypertension and obesity; these are also associated with the activation of circulating platelets and microvascular changes potentially reflective of endothelial dysfunction, which, as a precursor, may be an important contributor to the increased risk of CVD [32–35]. Correspondingly, recent studies, including a meta-analysis found that hyperuricemia increased the risk of coronary heart disease [36, 37]. Although the association between uric acid and CVD has been recognized for more than a century, it has been rediscovered and appreciated over the past 60 years, during which many epidemiologic studies were performed, most of these studies suggested that increased uric acid levels associate significantly with the risk of CVD [38–43]. Furthermore, several lines of experiments have produced evidence consistent with the results of epidemiological studies; specifically that uric acid is not an inert molecule but rather a deleterious factor that contributes greatly to the development of CVD [44–46]. In addition, uric acid appears to potentially induce persistent systemic and vascular inflammation, as well as vascular mechanisms that might contribute to the progression of atherosclerotic changes and stimulate prothrombotic activity; additionally, uric acid also influences the nitric oxide production, elevates blood pressure, and leads to endothelial dysfunction, potentially also increasing the risk of atherosclerosis [33, 47–53]. Uric acid, hyperuricemia and gout are normally observed with other risk factors that promote the occurrence of MI or CVD, and therefore the possible mechanism by which gout affects the risk of MI remains unclear.

### Clinical implications

To some extent, the findings from our meta-analysis of previous studies are clinically significant. Despite significant heterogeneity, all of the consistent evidence revealed an increased risk of MI in patients with gout. However, gout was only found to associate with non-fatal MI, but not fatal MI; this might largely be because of mortality was not attributable to the fatal MI events, which were excluded from the analysis, or because a lower incidence of these events was observed [16]. However, a recently published study that explored the risk of mortality from fatal MI in patients with gout also reported no relationship between gout and fatal MI [54]. The reason for this lack of association is also unclear and it might be attributable to classification bias and/or surveillance bias. Interestingly, neither study found an association between gout and risk of suffering from fatal MI or mortality from fatal MI, we believe this is well worth further exploration.

Gender-related differences in the association between gout and MI remain poorly understood. As gout predominantly affects men, and only a handful of studies have included women, and there remains controversy about the strength of gender-related associations [15–17, 55]. This controversy might result from differences in uric acid levels associated with physiological function, age, natural menopause and other baseline characteristics of the study cohorts [56]. However, the pooled results suggest that gout is associated with an increased risk of MI in both women and men. Interestingly, the result of our age groups analysis revealed an MI risk gradient that increased MI risk with a younger age at gout onset; in other words, the risk of MI was higher among young patients than older patients, and this finding was somewhat contradictory to the common theory that young people lack traditional CVD or MI risk factors. Although the reason for this phenomenon is unclear, the results of our meta-analysis suggest that these young patients with gout might have other confounding metabolic or vascular risk factors, although these potential factors were seldom observed or unknown; therefore, preventive management and monitoring of MI should be emphasized for all patients diagnosed with gout regardless of age.

### Limitations

This study has several important potential limitations inherent to the meta-analytic design. The crucial limitation of our study is the observational nature of the investigations, which might introduces bias from various sources, as well as confounding from other risk factors that could provide an alternative explanation for the significant association observed between gout and the risk of MI. However, allowing for the impossibility of a randomized controlled trial, the included cohort studies had an average NOS of 8, which might reduces the likelihood of recall bias. Admittedly, the NOS has a low inter-reviewer reliability, therefore, the quality appraisal should be performed by at least two reviewers to reduce assessment bias [57].

Second, our meta-analysis contained significant heterogeneity across studies in terms of the study design, follow-up duration, definition of gout, assessment of MI, as well as inconsistent adjustment for vascular risk factors in the multivariate analyses, which might have led to confounding bias; furthermore, inaccurate classification bias might also led to heterogeneity. Furthermore, the confidence intervals for the analysis of fatal MI were very wide, suggesting a possible association between gout and fatal MI that was simply not observed in this dataset, and additional studies will be needed to clarify this phenomenon. Other potential sources of bias are mainly attributable to the nature of data; most studies used data was from various healthcare databases, which usually lack modifying factors.

A third limitation is that there only five articles were confirmed and available for inclusion in this study, rendering the results prone to influence from publication bias or random error, and lacking sufficient reliability to draw a conclusion in a definitive manner.

Finally, although we searched without language restriction and used a comprehensive search strategy, some studies published in other journals or with negative findings might not appear in international journal databases and thus might not have been included in our meta-analysis.

## Conclusions

In conclusion, the results of this meta-analysis of five studies indicated that both male and female patients with gout have an increased risk of MI; however, this association remains with non-fatal MI, but not with fatal MI. Additionally, MI risk was found to increase in a gradient with a younger age at gout onset. Nevertheless, these results should be interpreted with caution, given the potential bias and confounding in the included studies.

## Supporting Information

S1 PRISMA Checklist PRISMA 2009 checklist used in this meta-analysis. (DOC)

S1 Table The covariates for adjustment in each study. (DOC)

S2 Table The Newcastle-Ottawa Scale (NOS) for assessing the methodological quality of included studies. (DOC)
