# Supplementary material for: Gout and Risk of Myocardial Infarction: A Systematic Review and Meta-Analysis of Cohort Studies
Source: PLoS One. 2015 Jul 31;10(7):e0134088. doi: 10.1371/journal.pone.0134088 (PMC4521845; doi:10.1371/journal.pone.0134088)
Supplement: S1 Table — (DOC) [file pone.0134088.s002.doc]

**Additional file S 1. The covariates for adjustment in each study.**

| **Author/year** | **Multivariate-adjusted RR** | **Adjustment for covariates** |
| --- | --- | --- |
| Krishnan/2006 [13] | 1.26 (1.14 to1.40) | Age, BMI, smoking, diastolic blood pressure, family history of AMI, total serum cholesterol, fasting blood glucose, serum creatinine, diuretic use, aspirin use, alcohol use, incident DM |
| Choi(Confirmed)/2007 [14] | 1.59 (1.04 to 2.41) | Age, BMI, smoking, physical activity, alcohol intake, family history of MI, history of hypertension, history of hypercholesterolemia, history of DM, aspirin use, diuretic use, total energy intake, trans fat, dietary cholesterol, protein, linoleic fatty acid, and the ratio of polyunsaturated fat to saturated fat |
| Choi(Self-reported)/2007 [14] | 1.51 (1.14 to 2.00) | Age, BMI, smoking, physical activity, alcohol intake, family history of MI, history of hypertension, history of hypercholesterolemia, history of DM, aspirin use, diuretic use, total energy intake, trans fat, dietary cholesterol, protein, linoleic fatty acid, and the ratio of polyunsaturated fat to saturated fat |
| De Vera(Men)/2010 [15] | 1.11 (0.99 to 1.23) | Age, baseline history of comorbidity medical condition (hypertension, diabetes, hyperlipidaemia and chronic obstructive pulmonary disease), baseline Charlson comorbidity score and monthly prescription drug use (non-steroidal anti-inflammatory drug, diuretic, statin, anticoagulant, aspirin, hormone replacement therapy and glucocorticoid) as time-varying covariates. |
| De Vera(Women)/2010 [15] | 1.39 (1.20 to 1.61) | Age, baseline history of comorbidity medical condition (hypertension, diabetes, hyperlipidaemia and chronic obstructive pulmonary disease), baseline Charlson comorbidity score and monthly prescription drug use (non-steroidal anti-inflammatory drug, diuretic, statin, anticoagulant, aspirin, hormone replacement therapy and glucocorticoid) as time-varying covariates |
| Kuo(Men)/2013 [16] | 1.33 (1.18 to 1.49) | Age, sex, histories of DM, hypertension, CHD, stroke and ESRD occurring between 1996 and 1999. |
| Kuo(Women)/2013 [16] | 1.21 (0.98 to1.48) | Age, sex, histories of DM, hypertension, CHD, stroke and ESRD occurring between 1996 and 1999. |
| Seminog(England)/2013 [17] | 1.82 (1.78 to 1.85) | Age, sex, time period in single calendar years and for region of residence and deprivation score associated with patient’s area of residence, in quintiles. |
| Seminog(ORLS)/2013 [17] | 1.95 (1.57 to 2.40) | Age, sex, in 5-year intervals, time period in single calendar years, district of residence. |

RR: relative risk, BMI: body mass index, DM: diabetes mellitus, CHD: coronary heart disease, MI: myocardial infarction, ESRD: end-stage renal disease
