# Supplementary material for: Gout and Risk of Myocardial Infarction: A Systematic Review and Meta-Analysis of Cohort Studies
Source: PLoS One. 2015 Jul 31;10(7):e0134088. doi: 10.1371/journal.pone.0134088 (PMC4521845; doi:10.1371/journal.pone.0134088)
Supplement: S2 Table — (DOC) [file pone.0134088.s003.doc]

| **Study** | **Selection** | | | | **Comparability** | **Outcome** | | | **Total score** |
| --- | --- | --- | --- | --- | --- | --- | --- | --- | --- |
|  | Exposed Cohort | Non exposed Cohort | Ascertainment of exposure | Outcome of interest |  | Assessment of outcome | Length of follow-up | Adequacy of follow-up |  |
| Krishnan/2006 [13] | * | * | * | * | ** | * | * | * | 9 |
| Choi( confirmed)/2007 [14] | - | * | * | * | ** | * | * | * | 8 |
| Choi(self-reported)/2007 [14] | - | * | - | * | ** | * | * | * | 7 |
| De Vera (men)/2010 [15] | * | * | * | - | ** | * | * | * | 8 |
| De Vera(women)/2010 [15] | * | * | * | - | ** | * | * | * | 8 |
| Kuo(men)/2013 [16] | * | * | * | - | ** | * | * | * | 8 |
| Kuo(women)/2013 [16] | * | * | * | - | ** | * | * | * | 8 |
| Seminog(England)/2013 [17] | * | * | * | - | ** | * | - | * | 7 |
| Seminog(ORLS)/2013 [17] | * | * | * | - | ** | * | * | * | 8 |

**Additional file 2** Methodological quality assessment (risk of bias) of included studies by Newcastle-Ottawa Scales
